# Uncovering a Hub Signaling Pathway of Antimicrobial-Antifungal-Anticancer Peptides’ Axis on Short Cationic Peptides via Network Pharmacology Study

**DOI:** 10.3390/ijms23042055

**Published:** 2022-02-12

**Authors:** Ki-Kwang Oh, Md. Adnan, Dong-Ha Cho

**Affiliations:** Department of Bio-Health Convergence, College of Biomedical Science, Kangwon National University, Chuncheon 24341, Korea; nivirna07@kangwon.ac.kr (K.-K.O.); mdadnan@kangwon.ac.kr (M.A.)

**Keywords:** short cationic peptides, antimicrobial peptides-antifungal peptides-anticancer peptides’ axis, HPIK, STAT3, HVTK, NOS2, HIF-1 signaling pathway

## Abstract

Short cationic peptides (SCPs) with therapeutic efficacy of antimicrobial peptides (AMPs), antifungal peptides (AFPs), and anticancer peptides (ACPs) are known as an enhancement of the host defense system. Here, we investigated the uppermost peptide(s), hub signaling pathway(s), and their associated target(s) through network pharmacology. Firstly, we selected SCPs with positive amino acid residues on N- and C- terminals under 500 Dalton via RStudio. Secondly, the overlapping targets between the bacteria-responsive targets (TTD and OMIM) and AMPs’ targets were visualized by VENNY 2.1. Thirdly, the overlapping targets between AFPs’ targets and fungal-responsive targets were exhibited by VENNY 2.1. Fourthly, the overlapping targets between cancer-related targets (TTD and OMIM) and fungal-responsive targets were displayed by VENNY 2.1. Finally, a molecular docking study (MDS) was carried out to discover the most potent peptides on a hub signaling pathway. A total of 1833 SCPs were identified, and AMPs’, AFPs’, and ACPs’ filtration suggested that 197 peptides (30 targets), 81 peptides (6 targets), and 59 peptides (4 targets) were connected, respectively. The AMPs―AFPs―ACPs’ axis indicated that 27 peptides (2 targets) were associated. Each hub signaling pathway for the enhancement of the host defense system was “Inactivation of Rap1 signaling pathway on AMPs”, “Activation of Notch signaling pathway on AMPs―AFPs’ axis”, and “Inactivation of HIF-1 signaling pathway on AMPs―AFPs―ACPs’ axis”. The most potent peptides were assessed via MDS; finally, HPIK on STAT3 and HVTK on NOS2 and on HIF-1 signaling pathway were the most stable complexes. Furthermore, the two peptides had better affinity scores than standard inhibitors (Stattic, 1400 W). Overall, the most potent SCPs for the human defense system were HPIK on STAT3 and HVTK on NOS2, which might inactivate the HIF-1 signaling pathway.

## 1. Introduction

Since the emergence of insulin application in the 1920s, peptide therapeutics have been revealed as highly selective, safe, efficacious, and well-tolerated pharmaceutical agents [1]. Peptides are intrinsic signaling molecules, possessing both biochemical and therapeutical attribution, and nearly more than 60 peptides are being used (FDA approved) worldwide as clinical medications [2]. Peptides’ critical properties as potential drug candidates are their high potency on target disease, specificity on a target protein, and minimal toxicity [3]. Certainly, peptides provide potential therapeutic intervention by binding to particular cell surface receptors, which stimulate intracellular effects. Given such unique and excellent characteristics, peptide drugs can be used as novel therapies or replacement therapies [4].

Bio-researchers have recently recognized the attractive pharmacological profile of short cationic peptides having significant antibacterial, antifungal, anticancer, and even immunomodulatory activities [5,6,7]. A report demonstrated that peptides with cation residues (Lysine, Arginine, Histidine) have more significant antimicrobial efficacy than peptides without cation residues [8]. Another study showed that short cationic peptides (SCPs; below six residues) expose better potency than longer peptides. Additionally, SCPs can be synthesized readily by following solid-phase peptide synthesis methods [9,10]. A pivotal property of cell-penetrating peptides (CPPs) is their cationic residues, facilitating permeability into the cell membrane [11]. Short peptides with cationic residues (Lysine, Arginine, Histidine) exist essentially in living organisms to function as antimicrobial activity [12]. In animals, antimicrobial peptides (AMPs) are often produced, acting as natural innate barriers and elevating immune response to combat microbial infection [13,14,15]. Interestingly, AMPs have tremendous therapeutic potential to function as antifungal peptides (AFPs) by suppressing the fungal growth such as *Candida* conidia and hyphae [16,17]. It implies that AMPs play essential roles in boosting the immune system against fungal attack and, hence, they are considered new biopharmaceuticals to fight or treat fungal infections. Recent studies have supported that cationic peptides act as immune modulators, recognizing signal molecules such as lipopolysaccharide secreted by bacterial or fungal molecules [18,19].

Evidence also suggests that AMPs demonstrate the antitumor activity by stimulating human cancer cells [20]. The constructed AMPs have positive amino acid residues that can bind effectively with negatively charged cancer cells’ components [21]. A study proved that AMPs can potentially disrupt the cancer cell membrane due to the strong electrostatic attraction present between positively charged AMPs and the negatively charged molecule “phosphatidylserine” on cancer cells’ plasma membranes [22]. Another report supports that AMPs activate the host immune defense system, working as anticancer peptides (ACPs) [23]. Despite these advantages, peptides have some intrinsic weaknesses, such as high molecular weight, degradability, and low permeability [24]. However, these limitations can be resolved through the traditional design of biotherapeutic peptides that are more suitable for use as convenient therapeutics. Multifunctional and useful cell-penetrating peptides offer more therapeutics and diagnostic merit, leading to the development of future medicines with improved target delivery, efficacy, and pharmacokinetic properties [25]. From these viewpoints, we used diverse multiple putative AMPs’ (or AFPs’) prediction tools to identify potential therapeutic of SCPs. The final peptides of ACPs were selected via public databases, and thus completed the AMPs―AFPs―ACPs’ axis on SCPs.

In this study, we performed a network pharmacology (NP) concept to achieve the AMPs―AFPs―ACPs’ axis. NP is a collective, systemic, and holistic approach to investigate the relation of molecule(s) and target(s), find the optimal molecule(s) on target protein(s), and provide a crucial hint for identifying the mechanism of a potential lead molecule(s) [26,27,28]. Moreover, Zhang B. et al. described that NP accelerates the decoding of TCM (Traditional Chinese Medicine) from an empirical-based therapy to an evidence-based therapy system, which improves modern drug discovery strategies [29].

**Figure 1:**
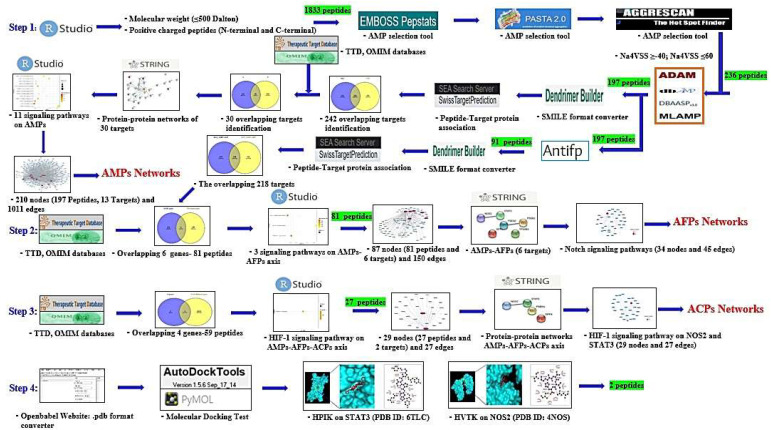
Workflow of AMPs―AFPs―ACPs’ axis analysis on network pharmacology. Green highlight: the number of peptides.

In our study, network pharmacology-based analysis was utilized to investigate triple therapeutic feasibility (AMPs―AFPs―ACPs’ axis) of SCPs. Firstly, SCPs (N- and C-terminal cationic groups; ≤500 Dalton) were selected via RStudio analysis. Commonly, N-terminal cationic groups contribute to the stabilization of the helical structure and C-terminal cationic groups can induce transduction into the cell membrane [30]. It implies that N- and C-terminal cationic groups are significant residues to penetrate the cell membrane. Secondly, the physicochemical propensity of selected SCPs was identified via AMPs’ screening platform, and a hub signaling pathway of AMPs between AMPs-related targets and host-responsive targets was analyzed. Thirdly, the AFPs’ screening platform was used to find AFPs from selected AMPs, and a hub signaling pathway of the AMPs―AFPs’ axis was identified between AFPs-related targets and host-responsive targets. Fourthly, the AMPs―AFPs―ACPs axis was constructed by retrieving cancer-related targets from public databases. Fifthly, SCPs accepted by the AMPs―AFPs―ACPs’ axis and targets on a hub signaling pathway were subjected to perform MDS. Finally, we found (via network pharmacology) a hub signaling of SCPs, which might be assumed to strengthen the host defense system. The workflow diagram is depicted in Figure 1.

## 2. Results

### 2.1. SCPs under 500 Dalton Rule 

The number of 1833 peptides with two sufficient conditions (positive N, C- terminals’ amino acid residues, under 500 Dalton rule [31]) was selected by RStudio analysis. In particular, ligands with less than 500 Dalton have a higher absorption and selectivity on targets in the drug development [32,33]. The selected peptides were enlisted (Supplementary Table S1).

### 2.2. Physicochemical Refinement for AMPs

The 1833 peptides were entered in EMBOSS Pepstats (https://www.ebi.ac.uk/Tools/seqstats/emboss_pepstats/) (Accessed on 2 May 2021) on Charge > 0 or 8 ≤ Isoelectric Point ≤ 12 [34]. Secondly, PASTA 2.0 (adjusted to “zero”) (https://protein.bio.unipd.it/) (Accessed on 2 May 2021)was utilized to predict the peptide aggregation propensity [35]. Thirdly, peptide aggregation was checked by AGGRESCAN (Na4VSS ≥ −40, Na4VSS ≤ 60) (http://bioinf.uab.es/aggrescan/) (Accessed on 4 May 2021), which was based upon aggregation propensity in vitro. Among 1833 peptides, the number of 236 peptides was selected (Supplementary Table S2). Fourthly, the 236 peptide sequences were input to four platforms including ADAM (http://bioinformatics.cs.ntou.edu.tw/adam/svm_tool.html) (Accessed on 6 May 2021), dbAMP (http://140.138.77.240/~dbamp/) (Accessed on 8 May 2021), DBAASP_v3.0_ (https://dbaasp.org/prediction/general) (Accessed on 11 May 2021), and MLAMP (http://www.jci-bioinfo.cn/MLAMP) (Accessed on 13 May 2021) to discover AMPs. Finally, from the four databases, 197 out of 236 peptides were obtained as suitable for AMPs (Supplementary Table S3).

### 2.3. AMPs’ Targets’ Identification

**Figure 2:**
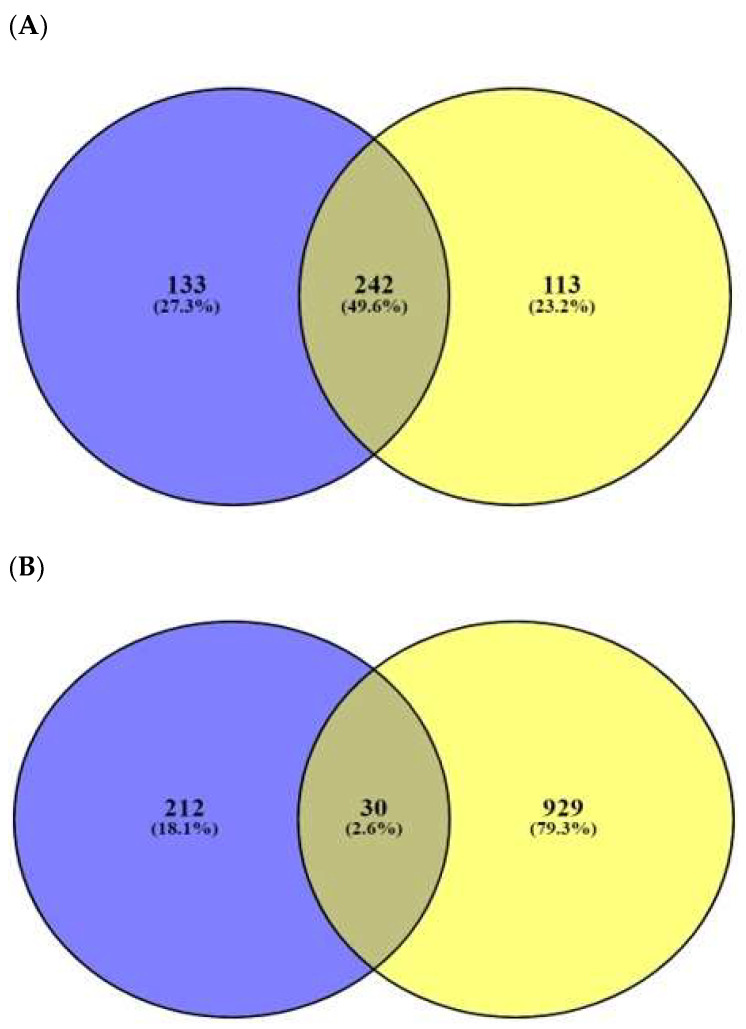
(**A**) The number of 242 overlapping targeted targets from SEA (375 targets) and STP (355 targets) on AMPs’ targets. (**B**) The number of 30 overlapping targets between the number of 242 overlapping targets and 959 bacterial respond targets.

The number of 197 peptides was converted into SMILE format via Dendrimer Builder (https://dendrimerbuilder.gdb.tools/) (Accessed on 16 May 2021). The SMILE format of peptide was input to the SEA (http://sea.bkslab.org/) (Accessed on 28 October 2021) and STP (http://www.swisstargetprediction.ch/) (Accessed on 18 May 2021) databases with “*Homo Sapiens”* setting. The number of 375 and 355 targets associated with the 197 peptides were identified by SEA and STP, respectively (Figure 2A), (Supplementary Table S4). The number of 242 overlapping targets was also identified from the two databases (Supplementary Table S5). Finally, the number of 30 targets overlapped between the number of 959 AMPs’ targets (extracted from the TTD and OMIM databases) (Figure 2B), (Table 1), (Supplementary Table S6), and the overlapping 242 targets were selected.

### 2.4. Signaling Pathways Responsive to Bacterial Infection on Human

**Figure 3:**
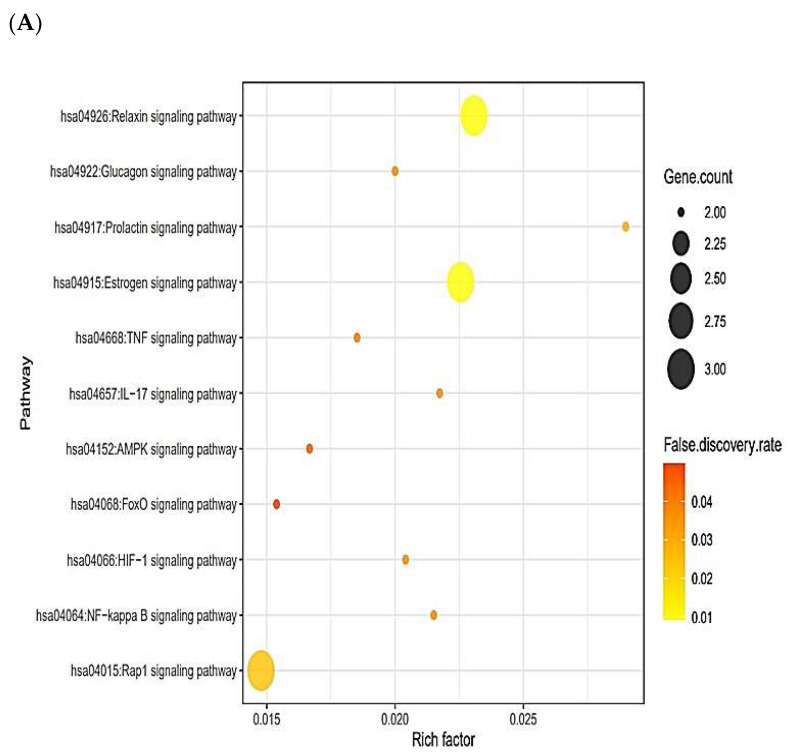

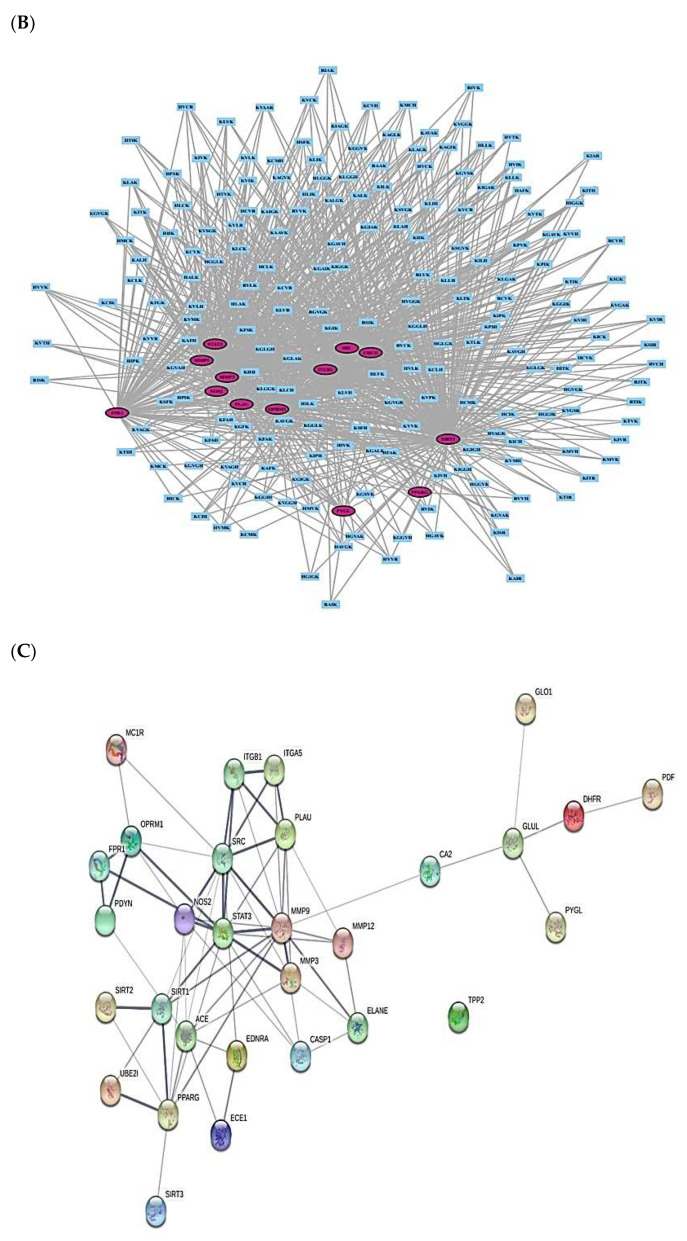

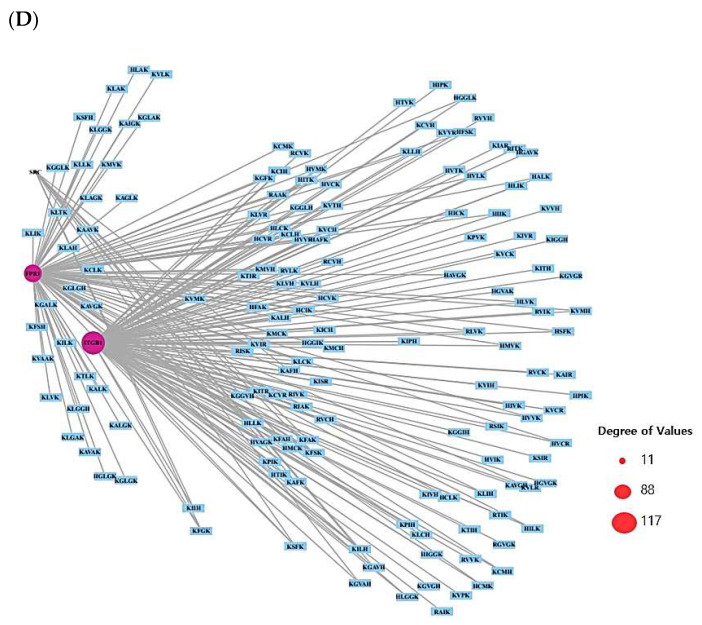
(**A**) The number of 11 signaling pathways on AMPs. (**B**) Networks of 11 signaling pathways on 210 nodes (197 Peptides, 13 Targets) and 1011 edges. (**C**) Protein–protein networks of 30 targets responded to bacterial infection. (**D**) Size map of Rap1 signaling pathway on SRC, FRC1, and ITGB1 targets (158 nodes and 216 edges).

The 13 out of the overlapping 30 targets were notably enriched in 11 signaling pathways via KEGG pathway enrichment analysis (Figure 3A). The targets of the 11 signaling pathways were enlisted (Table 2). The 13 targets were associated with the number of 197 peptides, and the constructed peptide–targets’ networks identified 210 nodes and 1011 edges (Figure 3B). The peptide–targets’ network analysis via the overlapping 30 targets was constructed by STRING, which indicated 30 nodes and 68 edges (Figure 3C). Among 11 signaling pathways, inactivation of Rap1 signaling pathway was identified as a hub signaling pathway through a bubble chart. Among 11 signaling pathways, the Rap1 signaling pathway’s targets were SRC, FPR1, and ITGB1, which were constructed with 158 nodes (3 targets, 155 peptides) and 216 edges on a size map (Figure 3D). Among the three targets (SRC, FPR1, and ITGB1), ITGB1 connected to 117 peptides was the highest degree of value. It implies that ITGB1 plays a vital role in Rap1 signaling pathways in human defense systems against bacterial infection.

### 2.5. Physicochemical Refinement for AFPs

The number of 197 peptides (AMPs) was input into AntipDS1_binary_model1, AntipDS1_binary_model2, and AntipDS1_binary_model3 in an antifungal peptide screening platform. Thereby, the number of 91 peptides was accepted by AFPs, which were defined as AMPs and AFPs with dual efficacy for enhancement of human defense system (Supplementary Table S7).

### 2.6. AFPs’ Targets’ Identification

**Figure 4:**
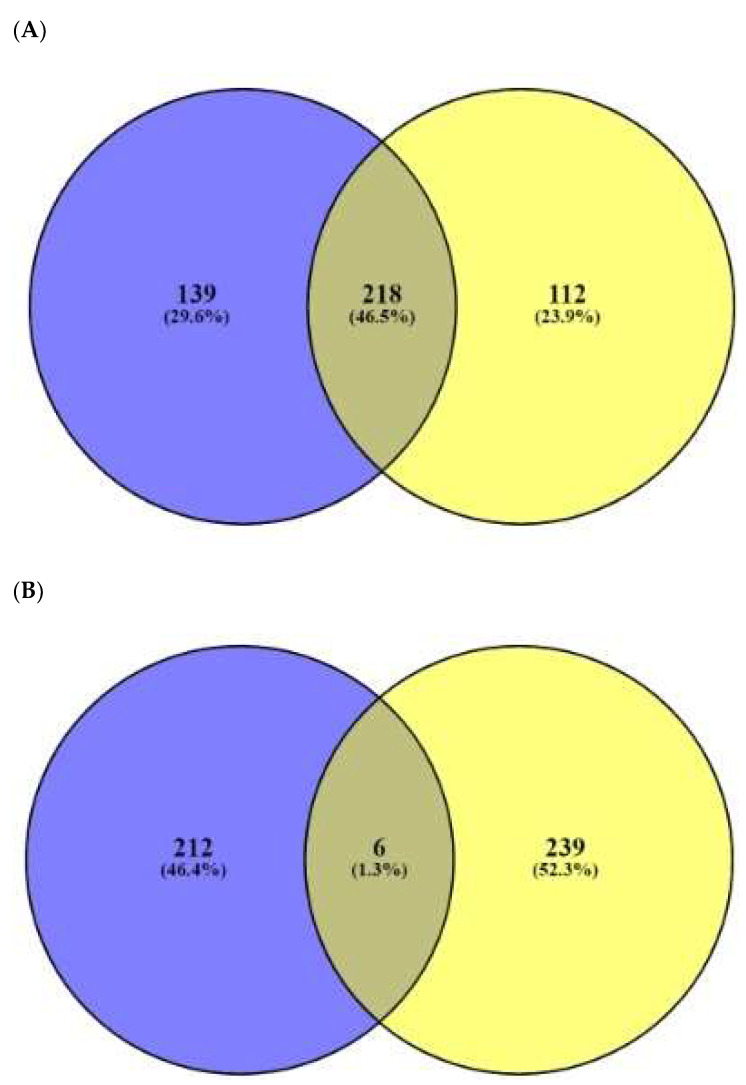
(**A**) The overlapping 218 targets identified by SEA (357 targets) and STP (330 targets) on AFPs’ targets. (**B**) The number of six overlapping targets between the number of 218 overlapping targets and 245 fungal-related targets.

The number of 91 peptides’ sequences was converted to SMILE format via Dendrimer Builder (https://dendrimerbuilder.gdb.tools/) (Accessed on 21 May 2021). The SMILE format of peptide was input to SEA (http://sea.bkslab.org/) (Accessed on 24 May 2021) and STP (http://www.swisstargetprediction.ch/) (Accessed on 26 May 2021) with “*Homo Sapiens”* setting. The numbers of 357 and 330 targets were identified from SEA and STP, respectively **(**Supplementary Table S8). Figure 4A displays that the number of 218 overlapping targets was selected from the two databases. (Supplementary Table S9). The number of six overlapping targets (TPSAB1, PSEN1, PSEN2, DPP4, STAT3, and NOS2) was identified between the number of AFPs’ targets (245 targets from the TTD and OMIM databases) (Figure 4B), (Supplementary Table S10) and the overlapping 218 targets.

### 2.7. Signaling Pathways Responsive to Fungal Infection on Human

**Figure 5:**
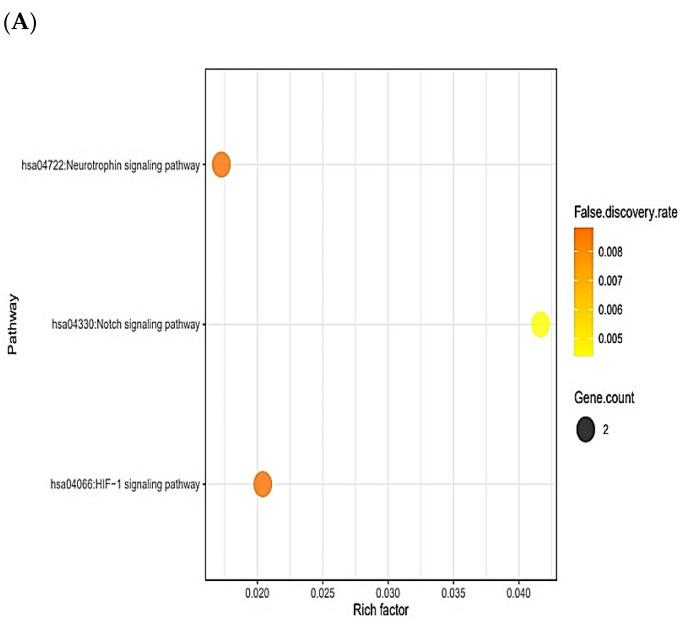

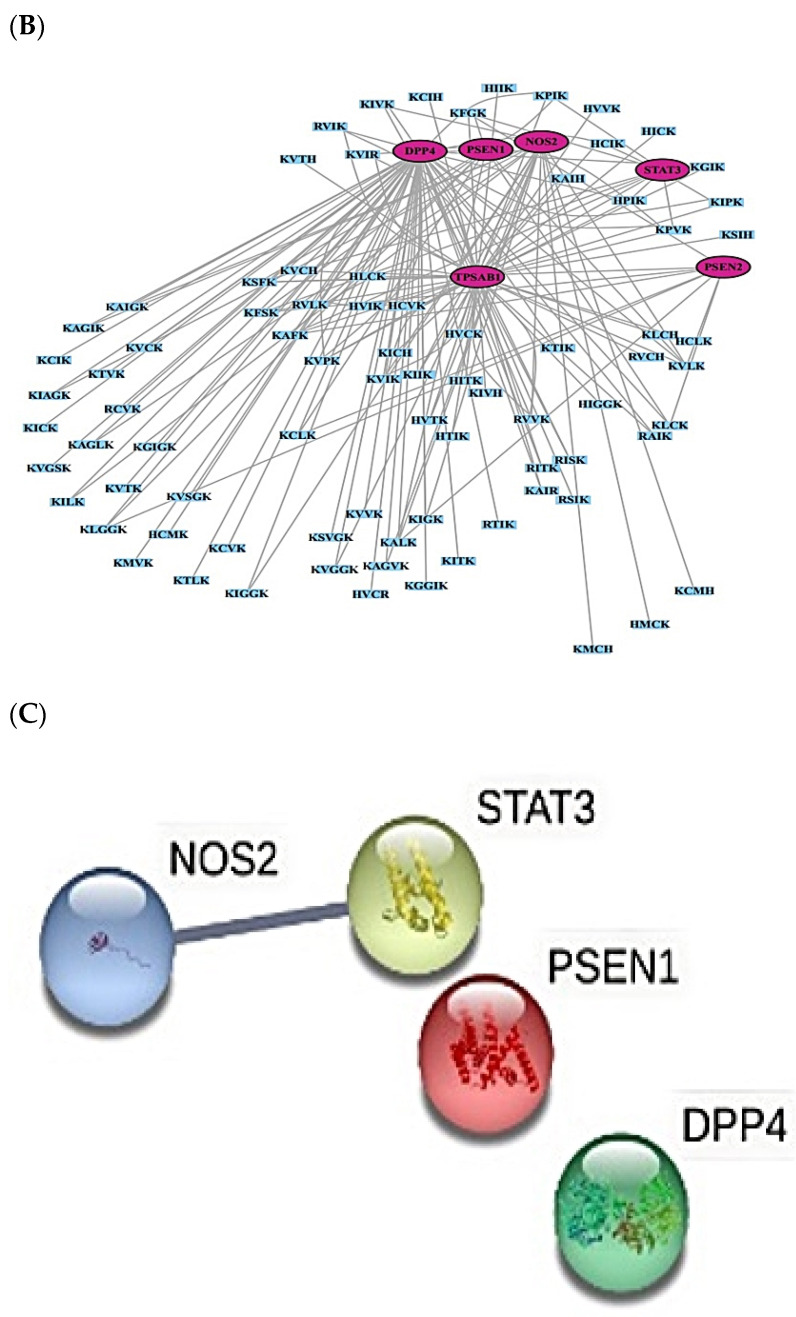

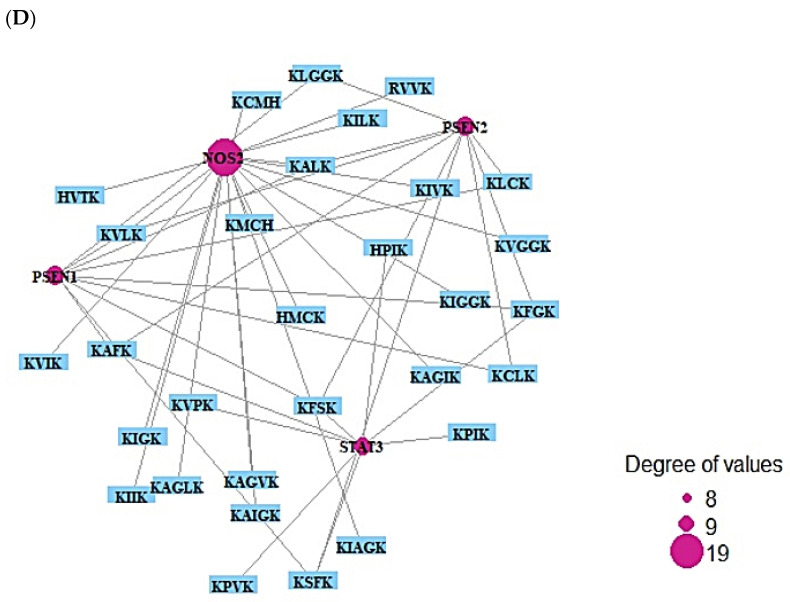
(**A**) The number of three signaling pathways on AMPs―AFPs’ axis. (**B**) Networks of three signaling pathways on 87 nodes (81 peptides and 6 targets) and 150 edges. (**C**) Protein–protein networks of AMPs―AFPs (6 targets). (**D**) Size map of Notch signaling pathways on NOS2, STAT3, PSEN1, and PSEN2 (34 nodes and 45 edges).

The six targets (TPSAB1, PSEN1, PSEN2, DPP4, STAT3, and NOS2) were connected to three signaling pathways via KEGG pathway enrichment analysis (Figure 5A). Table 3 shows the targets of the three signaling pathways. The six targets (TPSAB1, PSEN1, PSEN2, DPP4, STAT3, and NOS2) were related to the number of 81 peptides (Supplementary Table S11). The constructed network exposed 87 nodes (81 peptides, 6 targets) and 1011 edges (Figure 5B). The peptide–targets’ networking analysis via overlapping six targets (TPSAB1, PSEN1, PSEN2, DPP4, STAT3, and NOS2) was constructed by STRING, indicating six nodes and two edges (Figure 5C). Among three signaling pathways, activation of Notch signaling pathway was identified as a hub signaling pathway through a bubble chart. Notch signaling pathway’s targets were both PSEN1 and PSEN2, and their peptides–targets’ network was constructed on a size map (34 nodes and 45 edges) (Figure 5D). Among the four targets, PSEN1 and PSEN2 were connected to nine peptides (KLCK, KCLK, KALK, KVLK, KLGGK, KAFK, KFGK, KFSK, and KSFK), which might have more efficacy than any other AFPs. Additionally, it implies that both PSEN1 and PSEN2 play a pivotal role in the Notch signaling pathway of the human defense system against fungal infection on the AMPs―AFPs’ axis.

### 2.8. Cancer-Related Targets and ACPs’ Targets’ Identification

**Figure 6:**
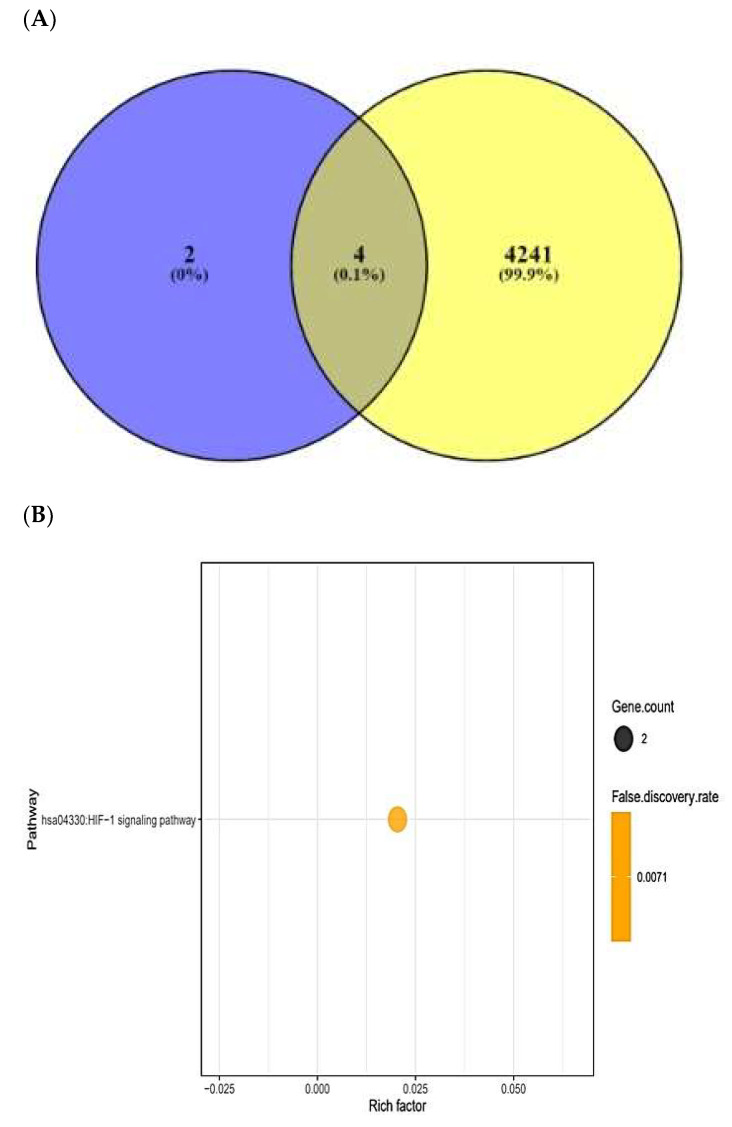

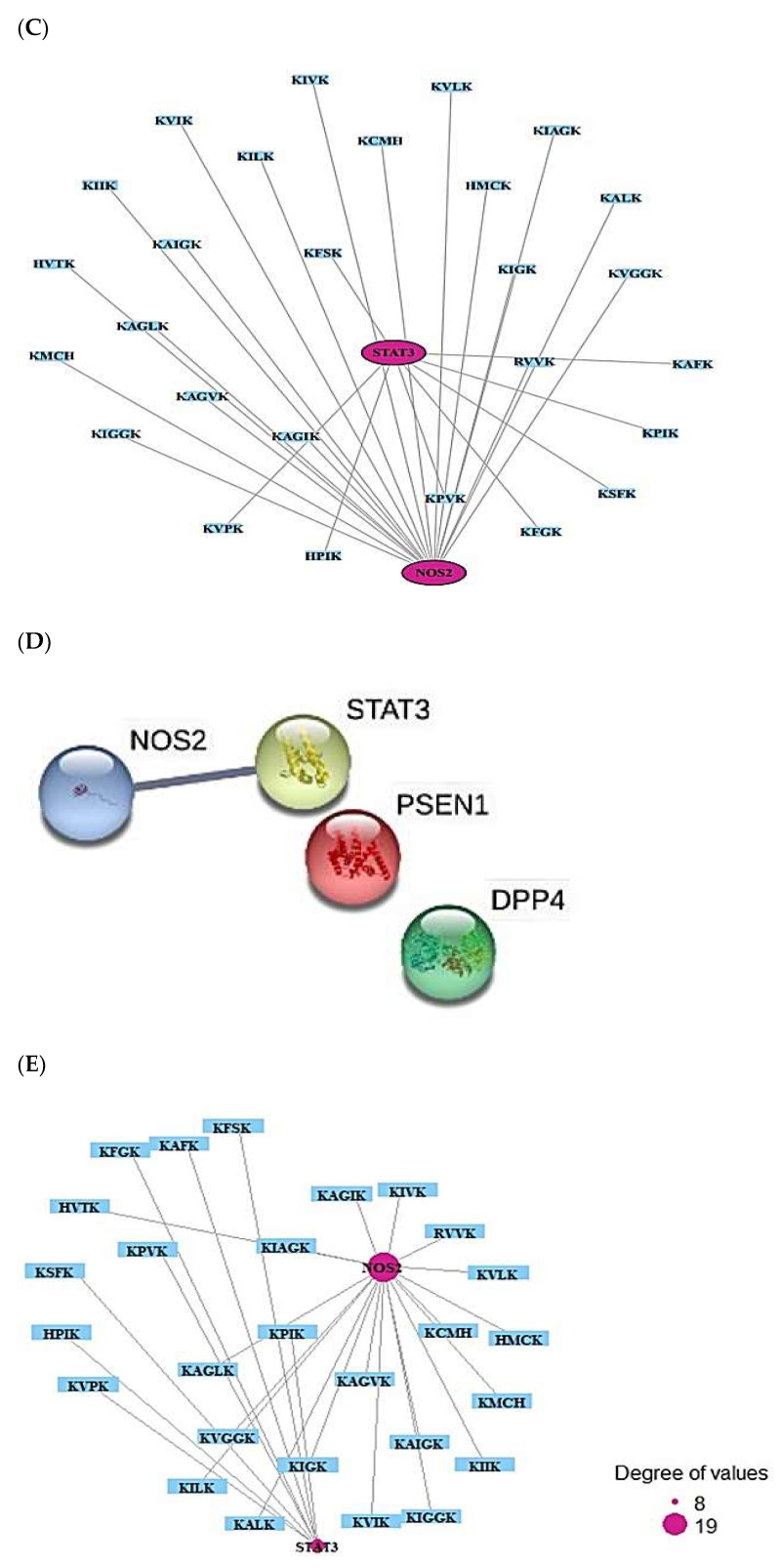
(**A**) Overlapping targets (four targets) between AMPs―AFPs’ axis (six targets) and cancer- related targets (4245 targets). (**B**) The number of one signaling pathway on AMPs―AFPs―ACPs’ axis. (**C**) Networks of HIF-1 signaling pathway on 29 nodes (27 peptides and 2 targets) and 27 edges. (**D**) Protein–protein networks of AMPs―AFPs―ACPs’ axis (four targets). (**E**) Size map of HIF-1 signaling pathway on NOS2 and STAT3 (29 nodes and 27 edges).

TTD and OMIM selected the number of 4247 cancer-related targets (Supplementary Table S12). The number of four out of six AFP-responsive targets was overlapped with the 4247 cancer-related targets (Figure 6A). The two targets (STAT3 and NOS2) were targeted to only HIF-1 signaling pathway via KEGG pathway enrichment analysis (Figure 6B), (Table 4). The two targets (STAT3 and NOS2) were related to the number of 27 peptides, and the constructed networks revealed 29 nodes (27 peptides, 2 targets) and 27 edges (Figure 6C). The peptide–targets’ networking analysis via overlapping four targets (PSEN1, DPP4, STAT3, NOS2) was constructed by STRING (six nodes and two edges) (Figure 6D). Only two targets (STAT3 and NOS2) were related directly to HIF-1 signaling pathway (Figure 6E). Both STAT3 and NOS2 targets were directly associated with HIF-1 signaling pathway, which played a crucial role in defending the cancer attack. The HIF-1 signaling pathway was connected particularly to all AMPs―AFPs―ACPs’ axes.

### 2.9. MDS on HIF-1 Signaling Pathway for Host Defense System

**Figure 7:**
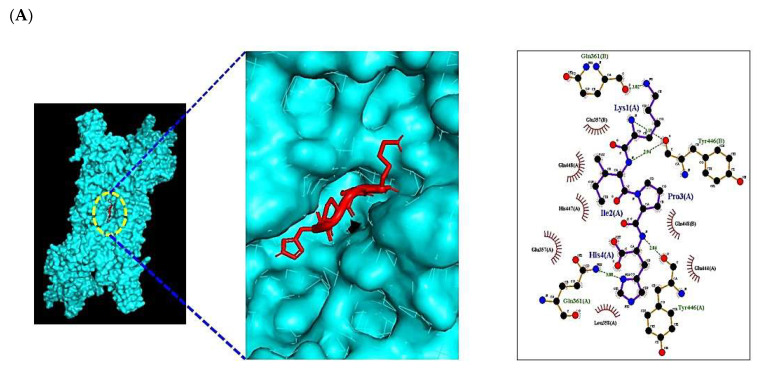

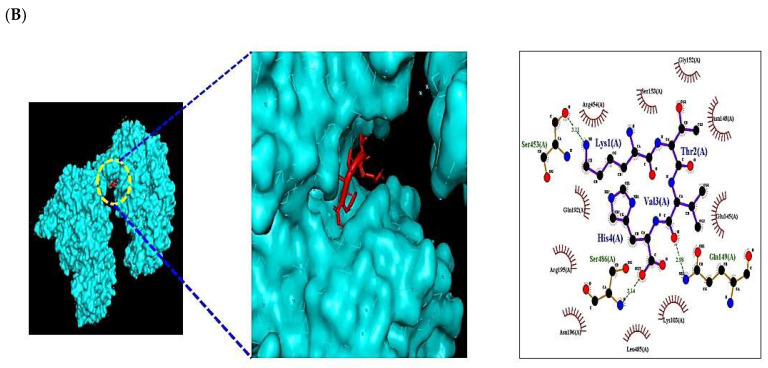
Molecular docking interaction between best-docked SCPs and targets. (**A**) HPIK on STAT3 (PDB ID: 6TLC). (**B**) HVTK on NOS2 (PDB ID: 4NOS).

The ultimate signaling pathway, HIF-1 signaling pathway, was connected to STAT3 (PDB ID: 6TLC) and NOS2 (PDB ID: 4NOS): The number of eight peptides (KPIK, KPVK, KVPK, HPIK, KAFK, KFGK, KSFK, and KFSK) was targeted to STAT3 target; additionally, the number of 19 peptides (RVVK, HMCK, KMCH, HVTK, KCMH, KIIK, KVIK, KILK, KVLK, KALK, KIVK, KIGK, KAIGK, KIAGK, KAGVK, KAGIK, KAGLK, KIGGK, and KVGGK) were targeted to NOS2 target. The physicochemical properties of the 27 peptides were profiled (Table 5). The number of eight peptides was targeted to STAT3 (PDB ID: 6TLC) and their priorities were as follows: HPIK (−7.3 kcal/mol), KAFK (−7.1 kcal/mol), KPIK (−7.0 kcal/mol), KPVK (−6.8 kcal/mol), KVPK (−6.8 kcal/mol), KFGK (−6.8 kcal/mol), KSFK (−6.7 kcal/mol), and KFSK (−6.4 kcal/mol). The “HPIK” peptide was the strongest affinity on STAT3 (PDB ID: 6TLC) in HIF-1 signaling pathway among eight peptides (Figure 7A) (Table 6). Likewise, 19 peptides were targeted to NOS2 (PDB ID: 4NOS). Their priorities were as follows: HVTK (−6.6 kcal/mol), KILK (−6.4 kcal/mol), KAGVK (−6.1 kcal/mol), KIGGK (−6.0 kcal/mol), KAGLK (−5.8 kcal/mol), KAIGK (−5.6 kcal/mol), HMCK (−5.5 kcal/mol), KIAGK (−5.5 kcal/mol), KVIK (−5.5 kcal/mol), KALK (−5.5 kcal/mol), RVVK (−5.4 kcal/mol), KIIK (−5.4 kcal/mol), KIVK (−5.4 kcal/mol), KMCH (−5.3 kcal/mol), KVGGK (−5.3 kcal/mol), KCMH (−5.1 kcal/mol), KVLK (−5.1 kcal/mol), and KAIGK (−5.0 kcal/mol). The “HVTK” peptide was the strongest affinity on NOS2 (PDB ID: 4NOS) in HIF-1 signaling pathway among 19 peptides (Figure 7B), (Table 7). This result showed that the uppermost promising peptides to strengthen the immune system against cancer were “HPIK” on STAT3 (PDB ID: 6TLC) and “HVTK” on NOS2 (PDB ID: 4NOS).

### 2.10. MDS of Positive Controls on HIF-1 Signaling Pathway

The greatest affinity peptide on STAT3 (PDB ID: 6TLC) was “HPIK” (−7.3 kcal/mol). A representative inhibitor of STAT3 is stattic (PubChem ID: 2779853), which interrupts the tumor cell growth by inhibiting lymphoma activity [36]. Thus, MDS of stattic (PubChem ID: 2779853) was selected to compare with “HPIK”. Consequently, the docking score of stattic (PubChem ID: 2779853) was −6.1 kcal/mol. The “HPIK” affinity on STAT3 (PDB ID: 6TLC) was better than stattic (PubChem ID: 2779853). The higher affinity peptide on NOS2 (PDB ID: 4NOS) was “HVTK” (−6.6 kcal/mol). A selective inhibitor of NOS2 is 1400 W (PubChem ID: 1433), which could inhibit U87MG cells (brain tumor cell) [37]. Hence, MDS of 1400 W (PubChem ID: 1433) was carried out to compare with “HVTK”; subsequently, the docking score of 1400 W (PubChem ID: 1433) was −5.2 kcal/mol.

## 3. Discussion

The SCPs were selected by two rigorous criteria: ≤500 Dalton and N-, C-terminal cationic amino acid residues. The number of 1833 SCPs was identified, and, consequently, 197 peptides (AMPs), 91 peptides (AMPs―AFPs’ axis), and 59 peptides (AMPs―AFPs―ACPs’ axis) were selected. The SCPs associated with signaling pathways were as follows: 197 peptides, 13 targets (AMPs); 81 peptides, 6 targets (AMPs―AFPs’ axis); and 27 peptides, 4 targets (AMPs―AFPs―ACPs’ axis). It was reported that SCPs have functioned as antimicrobial agents and host defense adjuvants [38]. A study suggested that TLR4 is an upregulated representative target in keratitis of bacterial infection, whereas SOD2 is an upregulated representative target in keratitis of fungal infection from Differentially Expressed Genes (DEGs) [39]. It implies that host responses against bacterial and fungal attack might induce significant differences in the immune system. Hence, we regarded it as an independent perturbation of the bacterial and fungal infection. A study indicated that AMPs could bind with negatively charged ions (phosphatidylserine) on the cancer cell membrane and trigger the host defense system [20]. Thus, we performed the analysis of AMPs―AFPs―ACPs’ axis to investigate potential SCPs for the host immune system. 

AMPs–targets’ network showed that the therapeutic efficacy of the host defense system was directly associated with 30 targets. The result of the KEGG pathway analysis of 30 targets indicated that 11 signaling pathways were connected to 13 out of 30 targets, suggesting that these signaling pathways were directly related to bacterial infection responses in the human immune system. 

The description of the 11 signaling pathways with bacterial infection were briefly discussed as follows. Relaxin signaling pathway: Relaxin prevents inflammatory cytokine induced by endotoxin in THP-1 (human monocytic cell line), which specializes the immune cells in the period of preterm birth [40]. Glucagon signaling pathway: Glucagon alleviates inflammatory responses of the airway due to association with the reduction of eosinophils and T lymphocytes by inhibiting TCD4+ cell proliferation [41,42]. Prolactin signaling pathway: Prolactin accelerates secretion of proinflammatory cytokines in peripheral immune cells, modulating the level of responses against pathogens [43,44]. Estrogen signaling pathway: Estrogen increases in the level of expression of AMPs in the host, thereby interrupting bacterial proliferation [45]. Additionally, estrogen stimulated the expression level of cell–cell junction proteins, thereby intensifying the epithelial rigidity and prohibiting unnecessary loss of outer cells during infection [46]. TNF signaling pathway: Tumor Necrosis Factor (TNF) can induce the recruitment of inflammatory cells and control the mechanism of antimicrobial activities [47]. It implies that TNF can work as a buffer element for immunopotentiation. IL-17 signaling pathway: The knockout groups of IL-17 are more highly susceptible to *K. pneumonia* infection than are the IL-17 expression groups [48]. AMPK signaling pathway: Activation of AMPK improves the host defense system against bacterial infection. Moreover, AMPK is associated with the innate and adaptive immune system [49]. FoxO signaling pathway: FoxO1 protein is expressed by a bacterial infection, strengthening the epithelial barrier of host cells and inducing the recruitment of Tregs (Regulatory T Cells) to activate the antibacterial defense [50]. HIF-1 signaling pathway: HIF-1α activation in the hypoxic condition recruits inflammatory-associated cells such as macrophages, neutrophils, and dendritic cells as well as inducing offensive cytokine production under bacterial infection [51]. HIF-1 inhibition can be a good strategy to relieve the inflammation level induced by the bacterial attack in aspects of the host immune system. Rap1 signaling pathway: The inactivation of Rap1 in lymphocytes is a representative treatment against inflammatory disorders [52]. On AMPs’ signaling pathways, the key mechanism might inhibit the Rap1 signaling pathway selected based on the rich factor. 

AMPs―AFPs’ axis–target networks showed that the therapeutic efficacy of the host defense system was directly associated with six targets. The result of the KEGG pathway analysis of six targets was connected to three signaling pathways. Neurotrophin signaling pathway: Inflammation signals in microglial cells induce the secretion of neurotrophins that function as mediators of pain [53,54]. It implies that the neurotrophin signaling pathway’s inactivation might modulate inflammatory-related proteins’ expression level, thereby resolving host defense-induced inflammation. HIF-1 signaling pathway: The deletion of hypoxia-regulated targets are resistant to fungal infection; more importantly, the low-oxygen condition makes fungal virulence attenuate in murine models [55]. Thus, inactivation of HIF-1 might interrupt the fungal penetration and host immune system. Notch signaling pathway: the Notch system plays important roles in Th1 and Th2 cell differentiation, and Notch-mediated immune responses are related to T cell development [56]. It supports the idea that the activation of Notch signaling pathway contributes to enhancing the host defense system. On AMPs―AFPs’ axis signaling pathways, a key signaling pathway is to activate the Notch signaling pathway, which was identified based on the rich factor

AMPs―AFPs―ACPs’ axis–target networks exhibited that the therapeutic efficacy of the host defense system was directly associated with four targets. The result of the KEGG pathway analysis on four targets was connected to one signaling pathway. HIF-1 signaling pathway: HIF-1 overexpression contributes to tumor growth, angiogenesis, and metastasis. However, the overexpression is caused by an oxygen-depleted condition in tumor cells [57,58]. Furthermore, hypoxia creates severe conditions under resistance to cancer therapy such as radiation and medication, increasing tumor survival [59]. It suggests that inactivation of the HIF-1 signaling pathway is an optimal strategy for cancer therapy. This work focused on immunomodulatory activities of SCPs, which may improve immune defenses and provide key therapeutic agents from large-scale peptides. We performed the MDT to select promising peptide candidate(s) on the HIF-1 signaling pathway, and, hence, the standard molecules (stattic and 1400 W) were compared with them. Moreover, we suggested a hub signaling pathway (HIF-1 signaling pathway), two key SCPs (HPIK and HVTK), and two key targets (STAT3 and NOS2). This analysis collectively suggested an overlapping signaling pathway “HIF-1 signaling pathway” on AMPs, AMPs―AFPs’ axis, and AMPs―AFPs―ACPs’ axis. Therefore, the inactivation of the HIF-1 signaling pathway using two selected peptides is a feasible treatment strategy for enhancing the host defense system. 

## 4. Materials and Methods

### 4.1. The Selection of Peptides via RStudio

The standard peptides were selected with positive amino acids (Lysine, Arginine, Histidine) on both terminals (N-terminal, C-terminal) or less than 500 Dalton. The selection method of these species was based on RStudio.

### 4.2. AMP Evaluation and Prediction

The selected peptides were assessed for AMP evaluation utilizing in silico analysis. Firstly, EMBOSS Pepstats (https://www.ebi.ac.uk/Tools/seqstats/emboss_pepstats/) (Accessed on 11 June 2021) [60] were used to identify the physicochemical properties of peptides. Secondly, aggregation of peptides was filtered with rigor on both PASTA 2.0 (https://protein.bio.unipd.it/) (Accessed on 12 June 2021) [35] and AGGRESCAN (http://bioinf.uab.es/aggrescan/) (Accessed on 12 June 2021) [34]. Subsequently, final AMPs were selected by ADAM (http://bioinformatics.cs.ntou.edu.tw/adam/svm_tool.html) (Accessed on 13 June 2021) [61], dbAMP (http://140.138.77.240/~dbamp/) (Accessed on 13 June 2021) [62], DBAASP_v3.0_ (https://dbaasp.org/prediction/general) (Accessed on 13 June 2021) [63], and MLAMP (http://www.jci-bioinfo.cn/MLAMP) (Accessed on 13 June 2021) [64].

### 4.3. AFP Evaluation and Prediction

The final AMPs’ sequences with FASTA format were input to the Antifp database (https://webs.iiitd.edu.in/raghava/antifp/predict3.php) (Accessed on 15 June 2021) [65]. The final AFPs were selected by the classifier of AntipDS1_binary_model1, AntipDS1_binary_model2, and AntipDS1_binary_model3. 

### 4.4. The Conversion of SMILES Format

The sequences of the final selected AMPs and AFPs were converted to SMILES format through Dendrimer Builder (https://dendrimerbuilder.gdb.tools/) (Accessed on 16 June 2021) [66]. 

### 4.5. Identification of Peptide–Target Networks and Microbial-Related Targets in Database

Based on SMILES (format), targets related to selected peptides were extracted from both SEA (http://sea.bkslab.org/) (Accessed on 17 June 2021) [67] and STP (http://www.swisstargetprediction.ch/) (Accessed on 17 June 2021) [68] with “*Homo Sapiens*” setting. The overlapping targets in the peptide(s)–target(s) networks between SEA and STP were identified by VENNY 2.1 (https://bioinfogp.cnb.csic.es/tools/venny/) (Accessed on 19 June 2021) [69]. The bacterial-responsive targets on human were obtained with “bacterial/germ/bacilli” from both the TTD (http://db.idrblab.net/ttd/) (Accessed on 19 June 2021) [70] and OMIM (https://www.omim.org/) (Accessed on 20 June 2021) [71] databases. After that, the overlapping targets between peptide(s)–target(s) and bacterial-responsive targets were identified by VENNY 2.1 (https://bioinfogp.cnb.csic.es/tools/venny/) (Accessed on 21 June 2021). 

### 4.6. Bubble Chart of Signaling Pathway Analysis of Overlapping Targets between Peptide–Targets and Bacterial-Responsive Targets’ Network

The final overlapping targets’ (bacterial-responsive targets on humans) networks were visualized by STRING (https://string-db.org/) (Accessed on 21 June 2021) [72]. A bubble chart of the Kyoto Encyclopedia of Genes and Genomes (KEGG) pathway based on the final overlapping targets was constructed by RStudio.

### 4.7. Identification of Peptide–Targets’ Network and Fungal-Related Targets in Database

Based on SMILES, targets associated with selected peptides were identified via both SEA (http://sea.bkslab.org/) (Accessed on 22 June 2021) and STP (http://www.swisstargetprediction.ch/) (Accessed on 22 June 2021) with “*Homo Sapiens*” setting. The overlapping targets in peptide–target network between SEA and STP were identified by VENNY 2.1 (https://bioinfogp.cnb.csic.es/tools/venny/) (Accessed on 23 June 2021). The fungal targets associated with human were obtained from both TTD (http://db.idrblab.net/ttd/) (Accessed on 23 June 2021) and OMIM (https://www.omim.org/) (Accessed on 23 June 2021), entering as “fungal”. The overlapping targets between peptide–targets and fungal-related targets were identified by VENNY 2.1 (https://bioinfogp.cnb.csic.es/tools/venny/) (Accessed on 24 June 2021). 

### 4.8. Bubble Chart of Signaling Pathway Analysis of Overlapping Targets between Peptide–Targets and Fungal-Responsive Targets’ Network

The final overlapping targets’ (fungal-responsive targets on the human) construction was visualized by STRING (https://string-db.org/) (Accessed on 24 June 2021). A bubble chart of the Kyoto Encyclopedia of Genes and Genomes (KEGG) pathway based on the final overlapping targets was constructed by RStudio.

### 4.9. Identification of Peptide–Targets’ Network and Cancer-Related Targets in Database

Based on SMILES, targets associated with selected peptides were identified via both SEA (http://sea.bkslab.org/) (Accessed on 24 June 2021) and STP (http://www.swisstargetprediction.ch/) (Accessed on 24 June 2021) with “*Homo Sapiens*” setting. The cancer-related targets on human were obtained with “cancer/tumor/neoplasia/carcinoma” from TTD (http://db.idrblab.net/ttd/) (Accessed on 25 June 2021) and OMIM (https://www.omim.org/) (Accessed on 25 June 2021). The overlapping targets between peptide–targets and cancer-related targets were identified by VENNY 2.1 (https://bioinfogp.cnb.csic.es/tools/venny/) (Accessed on 25 June 2021). 

### 4.10. Bubble Chart of Signaling Pathway Analysis of Overlapping Targets between Peptide–Targets and Cancer-Related Targets 

The final overlapping targets (cancer-related targets on the human) construction was visualized by STRING (https://string-db.org/) (Accessed on 26 June 2021). RStudio constructed a bubble chart of the KEGG pathway based on the final overlapping targets.

### 4.11. Preparation for Docking of Peptide Molecules

The peptide molecules were converted into SMILES format from Dendrimer builder. The converted SMILES were again converted into .pdb format using Open Babel (http://www.cheminfo.org/Chemistry/Cheminformatics/FormatConverter/index.html) (Accessed on 27 June 2021) [73]. Finally, the converted .pdb peptide was converted into .pdbqt format through Autodock.

### 4.12. Preparation for Docking of Target Proteins and Positive Controls to Compare with Final Peptides

Two target proteins of cancer, i.e., STAT3 (PDB ID: 6TLC) and NOS2 (PDB ID: 4NOS), identified from STRING were converted into .pdbqt format (https://www.rcsb.org/) (Accessed on 28 June 2021) from .pdb format in order to test the affinity of ligands via Autodock (http://autodock.scripps.edu/) (Accessed on 28 June 2021) [74]. Subsequently, two positive controls, i.e., stattic (PubChem ID: 2779853) for STAT3 and 1400 W (PubChem ID: 1433) for NOS2, were converted into.pdb format from .sdf format to upload to Pymol, and each of the two positive controls was converted again into .pdbqt format to measure affinity through Autodock.

### 4.13. Peptide–Target Proteins’ Docking Test

The final peptides were docked on target proteins, processing autodock4 by setting up four energy ranges and eight exhaustiveness ranges as defaults to obtain 10 different poses of ligand molecules [75]. The 2D binding interactions were constructed through LigPlot+ v.2.2 (https://www.ebi.ac.uk/thornton-srv/software/LigPlus/) (Accessed on 30 June 2021) [76].

## 5. Conclusions

The uppermost SCPs of AMPs―AFPs―ACPs’ axis for immunopotentiation were firstly investigated through network pharmacology. The number of 1833 SCPs was funneled sequentially through a peptide screening platform; thereby, the numbers of 197 SCPs (AMPs) and 91 SCPs (AMPs―AFPs axis) were obtained. The number of 27 SCPs (AMPs―AFPs―ACPs’ axis) was obtained as the final promising peptides through cancer-related targets’ analysis. The 27 SCPs (AMPs―AFPs―ACPs’ axis) were connected to only the HIF-1 signaling pathway with HPIK-STAT3 and HVTK-NOS2. This analysis provided the network of two SCPs, two targets, and one signaling pathway for the host defense system. Consequently, the key findings on the AMPs―AFPs―ACPs’ axis could be a promising therapeutic strategy for cellular protection against immune disorders.

## Figures and Tables

**Table 1:** The number of 30 targets overlapped between 959 AMPs’ targets and the overlapping 242 targets.

No.	Targets	No.	Targets
1	ACE	16	CA2
2	ECE1	17	ITGB1
3	EDNRA	18	GLO1
4	MMP3	19	MC1R
5	SIRT1	20	OPRM1
6	SIRT2	21	PPARG
7	TPP2	22	PYGL
8	UBE2I	23	SRC
9	CASP1	24	PLAU
10	FPR1	25	ELANE
11	MMP9	26	STAT3
12	PDYN	27	NOS2
13	MMP12	28	GLUL
14	SIRT3	29	DHFR
15	PDF	30	ITGA5

**Table 2:** Targets in 11 signaling pathways’ enrichment related to AMPs.

KEGG ID	Description	Targets	False Discovery Rate
hsa04917	Prolactin signaling pathway	SRC, STAT3	0.0283
hsa04926	Relaxin signaling pathway	SRC, NOS2, MMP9	0.0093
hsa04915	Estrogen signaling pathway	SRC, OPRM1, MMP9	0.0093
hsa04657	IL-17 signaling pathway	MMP3, MMP9	0.0359
hsa04064	NF-kappa B signaling pathway	PLAU, UBE2I	0.0359
hsa04066	HIF-1 signaling pathway	STAT3, NOS2	0.0359
hsa04922	Glucagon signaling pathway	SIRT1, PYGL	0.0360
hsa04668	TNF signaling pathway	MMP3, MMP9	0.0389
hsa04152	AMPK signaling pathway	SIRT1, PPARG	0.0448
hsa04068	FoxO signaling pathway	STAT3, SIRT1	0.0496
hsa04015	Rap1 signaling pathway	SRC, ITGB1, FPR1	0.0243

**Table 3:** Targets in three signaling pathways’ enrichment related to AMPs―AFPs’ axis.

KEGG ID	Description	Targets	False Discovery Rate
hsa04330	Notch signaling pathway	PSEN1, PSEN2	0.0044
hsa04066	HIF-1 signaling pathway	NOS2, STAT3	0.0088
hsa04722	Neurotrophin signaling pathway	PSEN1, PSEN2	0.0088

**Table 4:** Targets in one signaling pathway enrichment related to AMPs―AFPs―ACPs’ axis.

KEGG ID	Description	Targets	False Discovery Rate
hsa04066	HIF-1 signaling pathway	NOS2, STAT3	0.0071

**Table 5:** The physicochemical properties of the final 27 peptides on AMPs―AFPs―ACPs’ axis.

No.	Peptide Sequence	Residue Mass	Targets	Charge	Isoelectric Point	Aggregation Propensity
		(Da)		(>0)	(8≤; ≥12)	(Na4VSS ≥ −40; Na4VSS ≤ 60)
1	KPIK	466.65	NOS2	2	10.8	−34.6
2	KPVK	452.62	NOS2	2	10.8	−39.1
3	KVPK	452.62	NOS2	2	10.8	−39.1
4	HPIK	475.62	NOS2	1.5	9.2	−36.6
5	KAFK	474.62	NOS2	2	10.8	−30.0
6	KFGK	460.60	NOS2	2	10.8	−40.0
7	KSFK	490.62	NOS2	2	10.8	−35.1
8	KFSK	490.62	NOS2	2	10.8	−35.1
9	RVVK	482.66	STAT3	2	11.7	−6.7
10	HMCK	499.68	STAT3	1.5	8.0	−36.1
11	KMCH	499.68	STAT3	1.5	8.0	−36.1
12	HVTK	465.58	STAT3	1.5	9.2	−37.7
13	KCMH	499.68	STAT3	1.5	8.0	−36.1
14	KIIK	482.70	STAT3	2	10.8	8.6
15	KVIK	468.67	STAT3	2	10.8	4.0
16	KILK	482.70	STAT3	2	10.8	−0.3
17	KVLK	468.67	STAT3	2	10.8	−4.8
18	KALK	440.61	STAT3	2	10.8	−37.4
19	KIVK	468.67	STAT3	2	10.8	4.0
20	KIGK	426.59	STAT3	2	10.8	−38.6
21	KAIGK	497.57	STAT3	2	10.8	−19.0
22	KIAGK	497.57	STAT3	2	10.8	−19.0
23	KAGVK	483.64	STAT3	2	10.8	−23.6
24	KAGIK	497.67	STAT3	2	10.8	−19.0
25	KAGLK	497.67	STAT3	2	10.8	−27.8
26	KIGGK	483.65	STAT3	2	10.8	−29.0
27	KVGGK	469.62	STAT3	2	10.8	−33.5

**Table 6:** Binding energy and interactions of potential active SCPs and standard molecule (stattic) on STAT3 (PDB ID: 6TLC).

**Peptide Sequence**	**Binding Energy (kcal/mol)**	**Hydrogen Bond Interactions**	**Hydrophobic Interactions**
		**Amino Acid Residue**	**Amino Acid Residue**
HPIK	−7.3	Gln361, Tyr446	Gln448, Glu444, Leu358
			Glu357, His447, Gln448
KAFK	−7.1	Lys363, Thr443, Tyr446,	Gln448, Glu357, His447
		Glu357, Gly449, Gln448,	Val445, Glu444
		Gln361	
KPIK	−7.0	Gln361, Tyr446, Gln361	Glu444, Val445, Gly449,
			Gln448, Glu357, Gln448,
			Leu362
KPVK	−6.8	Glu444, Tyr446, Gln361,	Gln448, Glu357, Leu358,
		Tyr446	Gly449, Val445
KVPK	−6.8	Gln361	His447 Glu357, Gln448,
			Tyr446, Gly449, Val445
KFGK	−6.8	Glu306, Arg278, Lys282,	Ile309, Tyr360, Lys283,
		Gln361, Gln448	Gln279, Glu286, Leu362,
			Lys363, Leu450, Gly449,
			Val310
KSFK	−6.7	Lys363, Gly449, Gln448	Glu444, His447, Tyr446,
		Tyr446, Gln361, Thr443	His447, Glu357, Gln448
			Val445, Glu357, Leu358
KFSK	−6.4	Tyr446, Gly449, Gln361	Gln448, Glu444, Val445
		Gln448	His447, Glu357, His447,
			Glu357
**Compound (PubChem ID)**	**Binding energy (kcal/mol)**	**Hydrogen Bond Interactions**	**Hydrophobic Interactions**
		**Amino Acid Residue**	**Amino Acid Residue**
Stattic (2779853)	−6.1	Gly449, Tyr446, Gln361	Gln448, Tyr446, Glu357,
			His447

**Table 7:** Binding energy and interactions of potential active SCPs and standard molecule (1400 W) on NOS2 (PDB ID: 4NOS).

**Peptide Sequence**	**Binding Energy (kcal/mol)**	**Hydrogen Bond Interactions**	**Hydrophobic Interactions**
		**Amino Acid Residue**	**Amino Acid Residue**
HVTK	−6.6	Gln149, Ser486, Ser453	Asn148, Glu145, Lys103
			Leu485, An196, Arg195,
			Gln192, Arg454, Ser153,
			Gly152
KILK	−6.4	Ser486, Gln149, Glu145	Lys105, ALa104, Leu485,
			Lys103, Gln192, Gly152,
			Leu100, Ser153, Pro273
KAGVK	−6.1	Ser153, Gln192, Asn196,	Gly152, Arg195, Gln149,
		Arg454, Ser453	Ser486, Leu485, Glu145,
			Lys103, Leu100
KIGGK	−6.0	Glu450, Arg454, Asn196,	Ser453, Trp206, Leu100,
		Lys103, Gln192, Arg195	Leu485, Ser486, Gly152,
			Gln149, Ser153
KAGLK	−5.8	Gln149, Gln192, Asn148,	Arg454, Arg195, Gly275,
		Glu145	Asp274, Pro273, Gly152,
			Ser486, Lys103, Phe188,
			Leu485, Leu100
KAIGK	−5.6	Arg454, Ser486, Gln192	Gln149, Lys103, Leu485,
			Leu100, Glu145, Asn148,
			Gly152
HMCK	−5.5	Gln192, Ser486, Glu245	Ser153, Gly152, Arg195,
		Asn196, Arg454	Lys103, Leu485, Gln149,
			Leu100, Ser453
KIAGK	−5.5	Glu145, Gln192, Arg195	Lys103, Leu100, Leu485,
			Arg454, Gln149, Asn148
KVIK	−5.5	Glu145, Asn196, Gln192,	Ser486, Leu485, Gln149,
		Arg454	Asn148, Pro273, Lys103
KALK	−5.5	Ser486, Gln149, Asp274	Leu100, Gly152, Pro273,
			Asn148, Glu145, Lys103,
			Leu485
RVVK	−5.4	Gln192	Ser153, Arg454, Gln149,
			Gly152, Asp274, Asn148,
			Lys103, Leu485, Leu100
KIIK	−5.4	Lys103, Gln192, Arg195	Leu485, Gln149, Leu100,
			Ser453, Ser153, Gly152,
			Glu145, Ser486
KIVK	−5.4	Pro273, An148, Glu145	Ser486, Lys103, Leu485,
			Asn196, Leu100, Arg454,
			Gln149, Gly152
KMCH	−5.3	Arg195, Gln192, Ser486,	Ser153, Asp274, Asn148,
		Gln149, Lys103	Gly275, Pro273, Glu145,
			Leu100, Arg454
KVGGK	−5.3	Thr121, Arg86, Thr126	Trp90, Glu479, Ile119,
			Val85, Arg83, His84,
			Leu116, Thr109, Pro122,
			Lys123
KIGK	−5.3	Gly152, Lys103, Glu145,	Asn148, Leu485, Ser486,
		Asn196, Arg454	Leu100, Gln149, Ser453
			Ser153
KCMH	−5.1	Lys103, Gln149, Gln192	Ser453, Gly152, Leu100,
			Leu485, Ser486, Ser153
KVLK	−5.1	Ile277, Asn390, Gly279	Arg278, Ser276, Leu344,
			Arg301, Ile391, Pro281,
			Tyr389, Arg388
KAGIK	−5.0	Asn196	Arg454, Arg195, Ser153,
			Leu100, Lys103, Ser486,
			Leu485, Glu145, Asn148,
			Gln149, Gly152, Gln192
**Compound (PubChem ID)**	**Binding energy (kcal/mol)**	**Hydrogen Bond Interactions**	**Hydrophobic Interactions**
		**Amino Acid Residue**	**Amino Acid Residue**
1400 W(1433)	−5.2	Gln97	Gly455, Arg452, Tyr451,
			Met94, Gln448, Thr95,
			Phe96

## Data Availability

All data generated or analyzed during this study are included in this published article (and its Supplementary Information files).

## Supplementary Materials

The following supporting information can be downloaded at: https://www.mdpi.com/article/10.3390/ijms23042055/s1.

## Abbreviations

ACPs	Anticancer peptides
AFPs	Antifungal peptides
AMPs	Antimicrobial peptides
AMPK	AMP-activated protein kinase
DEGs	Differentially Expressed Genes
FDA	Food and Drug Administration
FoxO	Forkhead box O
HIF-1	Hypoxia Inducible Factor 1
KEGG	Kyoto Encyclopedia of Genes and Genomes
MDS	Molecular Docking Study
Rap1	Repressor Activator Protein
SCPs	Short cationic peptides
SEA	Similarity Ensemble Approach
SMILES	Simplified Molecular Input Line Entry System
STP	SwissTargetPrediction
TCM	Traditional Chinese Medicine
TNF	Tumor Necrosis Factor
Tregs	Regulatory T cells

## References

[1] Uhlig T., Kyprianou T., Martinelli F.G., Oppici C.A., Heiligers D., Hills D., Calvo X.R., Verhaert P. (2014). The emergence of peptides in the pharmaceutical business: From exploration to exploitation. EuPA Open Proteom..

[2] Lau J.L., Dunn M.K. (2018). Therapeutic peptides: Historical perspectives, current development trends, and future directions. Bioorg. Med. Chem..

[3] Angell Y., Holford M., Moos W.H. (2018). Building on Success: A Bright Future for Peptide Therapeutics. Protein Pept. Lett..

[4] Fosgerau K., Hoffmann T. (2015). Peptide therapeutics: Current status and future directions. Drug Discov. Today.

[5] Bhat Z.F., Kumar S., Bhat H.F. (2015). Bioactive peptides of animal origin: A review. J. Food Sci. Technol..

[6] Mishra B., Wang X., Lushnikova T., Zhang Y., Golla R.M., Narayana J.L., Wang C., McGuire T.R., Wang G. (2018). Antibacterial, antifungal, anticancer activities and structural bioinformatics analysis of six naturally occurring temporins. Peptides.

[7] Haney E.F., Hancock R.E.W. (2013). Peptide design for antimicrobial and immunomodulatory applications. Biopolymers.

[8] Bormann N., Koliszak A., Kasper S., Schoen L., Hilpert K., Volkmer R., Kikhney J., Wildemann B. (2017). A short artificial antimicrobial peptide shows potential to prevent or treat bone infections. Sci. Rep..

[9] Kuppusamy R., Willcox M., Black D.S.C., Kumar N. (2019). Short cationic peptidomimetic antimicrobials. Antibiotics.

[10] Wenzel M., Chiriac A.I., Otto A., Zweytick D., May C., Schumacher C., Gust R., Albada H.B., Penkova M., Krämer U. (2014). Small cationic antimicrobial peptides delocalize peripheral membrane proteins. Proc. Natl. Acad. Sci. USA.

[11] Ziegler A., Nervi P., Dürrenberger M., Seelig J. (2005). The cationic cell-penetrating peptide CPPTAT derived from the HIV-1 protein TAT is rapidly transported into living fibroblasts: Optical, biophysical, and metabolic evidence. Biochemistry.

[12] Hancock R.E.W., Lehrer R. (1998). Cationic peptides: A new source of antibiotics. Trends Biotechnol..

[13] Brogden K.A., Ackermann M., McCray P.B., Tack B.F. (2003). Antimicrobial peptides in animals and their role in host defences. Int. J. Antimicrob. Agents.

[14] Oppenheim J.J., Yang D. (2005). Alarmins: Chemotactic activators of immune responses. Curr. Opin. Immunol..

[15] Bowdish D., Davidson D., Hancock R. (2005). A Re-evaluation of the Role of Host Defence Peptides in Mammalian Immunity. Curr. Protein Pept. Sci..

[16] van Eijk M., Boerefijn S., Cen L., Rosa M., Morren M.J.H., van der Ent C.K., Kraak B., Dijksterhuis J., Valdes I.D., Haagsman H.P. (2020). Cathelicidin-inspired antimicrobial peptides as novel antifungal compounds. Med. Mycol..

[17] Netea M.G., Joosten L.A.B., Van Der Meer J.W.M., Kullberg B.J., Van De Veerdonk F.L. (2015). Immune defence against Candida fungal infections. Nat. Rev. Immunol..

[18] Hancock R.E.W., Sahl H.G. (2006). Antimicrobial and host-defense peptides as new anti-infective therapeutic strategies. Nat. Biotechnol..

[19] Finlay B.B., Hancock R.E.W. (2004). Can innate immunity be enhanced to treat microbial infections?. Nat. Rev. Microbiol..

[20] Deslouches B., Peter Di Y. (2017). Antimicrobial peptides with selective antitumor mechanisms: Prospect for anticancer applications. Oncotarget.

[21] Gaspar D., Salomé Veiga A., Castanho M.A.R.B. (2013). From antimicrobial to anticancer peptides. A review. Front. Microbiol..

[22] Digumarti R., Bapsy P.P., Suresh A.V., Bhattacharyya G.S., Dasappa L., Shan J.S., Gerber D.E. (2014). Bavituximab plus paclitaxel and carboplatin for the treatment of advanced non-small-cell lung cancer. Lung Cancer.

[23] Guzmán-Rodríguez J.J., Ochoa-Zarzosa A., López-Gómez R., López-Meza J.E. (2015). Plant antimicrobial peptides as potential anticancer agents. BioMed Res. Int..

[24] Lee A.C.L., Harris J.L., Khanna K.K., Hong J.H. (2019). A comprehensive review on current advances in peptide drug development and design. Int. J. Mol. Sci..

[25] Xie J., Bi Y., Zhang H., Dong S., Teng L., Lee R.J., Yang Z. (2020). Cell-Penetrating Peptides in Diagnosis and Treatment of Human Diseases: From Preclinical Research to Clinical Application. Front. Pharmacol..

[26] Oh K.K., Adnan M., Cho D.H. (2020). Active ingredients and mechanisms of Phellinus linteus (grown on *Rosa multiflora*) for alleviation of Type 2 diabetes mellitus through network pharmacology. Gene.

[27] Oh K.K., Adnan M., Cho D.H. (2020). Network pharmacology approach to bioactive chemical compounds identified from Lespedeza bicolor lignum methanol extract by GC–MS for amelioration of hepatitis. Gene Rep..

[28] Oh K.K., Adnan M., Cho D.H. (2020). Network pharmacology of bioactives from Sorghum bicolor with targets related to diabetes mellitus. PLoS ONE.

[29] Li S., Zhang B. (2013). Traditional Chinese medicine network pharmacology: Theory, methodology and application. Chin. J. Nat. Med..

[30] Pescina S., Ostacolo C., Gomez-Monterrey I.M., Sala M., Bertamino A., Sonvico F., Padula C., Santi P., Bianchera A., Nicoli S. (2018). Cell penetrating peptides in ocular drug delivery: State of the art. J. Control. Release.

[31] Lipinski C.A., Lombardo F., Dominy B.W., Feeney P.J. (2001). Experimental and computational approaches to estimate solubility and permeability in drug discovery and development settings. Adv. Drug Deliv. Rev..

[32] Schultes S., De Graaf C., Haaksma E.E.J., De Esch I.J.P., Leurs R., Krämer O. (2010). Ligand efficiency as a guide in fragment hit selection and optimization. Drug Discov. Today Technol..

[33] Leeson P.D., Springthorpe B. (2007). The influence of drug-like concepts on decision-making in medicinal chemistry. Nat. Rev. Drug Discov..

[34] Lee J.H., Chung H., Shin Y.P., Kim I.W., Natarajan S., Veerappan K., Seo M., Park J., Hwang J.S. (2020). Transcriptome analysis of psacothea hilaris: De novo assembly and antimicrobial peptide prediction. Insects.

[35] Walsh I., Seno F., Tosatto S.C.E., Trovato A. (2014). PASTA 2.0: An improved server for protein aggregation prediction. Nucleic Acids Res..

[36] Scuto A., Kujawski M., Kowolik C., Krymskaya L., Wang L., Weiss L.M., DiGiusto D., Yu H., Forman S., Jove R. (2011). STAT3 inhibition is a therapeutic strategy for ABC-like diffuse large B-cell lymphoma. Cancer Res..

[37] Palumbo P., Lombardi F., Augello F.R., Giusti I., Luzzi S., Dolo V., Cifone M.G., Cinque B. (2019). NOS2 inhibitor 1400 W induces autophagic flux and influences extracellular vesicle profile in human glioblastoma U87MG cell line. Int. J. Mol. Sci..

[38] Brown K.L., Hancock R.E.W. (2006). Cationic host defense (antimicrobial) peptides. Curr. Opin. Immunol..

[39] Tian R., Zou H., Wang L., Liu L., Song M., Zhang H. (2020). Analysis of differentially expressed genes in bacterial and fungal keratitis. Indian J. Ophthalmol..

[40] Horton J.S., Yamamoto S.Y., Bryant-Greenwood G.D. (2011). Relaxin modulates proinflammatory cytokine secretion from human decidual macrophages. Biol. Reprod..

[41] Insuela D.B.R., Azevedo C.T., Coutinho D.S., Magalhães N.S., Ferrero M.R., Ferreira T.P.T., Cascabulho C.M., Henriques-Pons A., Olsen P.C., Diaz B.L. (2019). Glucagon reduces airway hyperreactivity, inflammation, and remodeling induced by ovalbumin. Sci. Rep..

[42] Long H., Liao W., Wang L., Lu Q. (2016). A Player and Coordinator: The Versatile Roles of Eosinophils in the Immune System. Transfus. Med. Hemotherapy.

[43] Boutet P., Sulon J., Closset R., Detilleux J., Beckers J.F., Bureau F., Lekeux P. (2007). Prolactin-induced activation of nuclear factor κB in bovine mammary epithelial cells: Role in chronic mastitis. J. Dairy Sci..

[44] Yu-Lee L.Y. (2002). Prolactin modulation of immune and inflammatory responses. Recent Prog. Horm. Res..

[45] Hannan T.J., Hooton T.M., Hultgren S.J. (2013). Estrogen and recurrent UTI: What are the facts?. Sci. Transl. Med..

[46] Lüthje P., Brauner H., Ramos N.L., Övregaard A., Gläser R., Hirschberg A.L., Aspenström P., Brauner A. (2013). Estrogen supports urothelial defense mechanisms. Sci. Transl. Med..

[47] Ehlers S. (2003). Role of tumour necrosis factor (TNF) in host defence against tuberculosis: Implications for immunotherapies targeting TNF. Ann. Rheum. Dis..

[48] Qian Y., Kang Z., Liu C., Li X. (2010). IL-17 signaling in host defense and inflammatory diseases. Cell. Mol. Immunol..

[49] Silwal P., Kim J.K., Yuk J.M., Jo E.K. (2018). AMP-Activated protein kinase and host defense against infection. Int. J. Mol. Sci..

[50] Graves D.T., Milovanova T.N. (2019). Mucosal Immunity and the FOXO1 Transcription Factors. Front. Immunol..

[51] Palazon A., Goldrath A.W., Nizet V., Johnson R.S. (2014). HIF Transcription Factors, Inflammation, and Immunity. Immunity.

[52] Mor A., Haklai R., Ben-Moshe O., Mekori Y.A., Kloog Y. (2011). Inhibition of contact sensitivity by farnesylthiosalicylic acid-amide, a potential rap1 inhibitor. J. Investig. Dermatol..

[53] Heese K., Hock C., Otten U. (1998). Inflammatory signals induce neurotrophin expression in human microglial cells. J. Neurochem..

[54] Mannion R.J., Costigan M., Decosterd I., Amaya F., Ma Q.P., Holstege J.C., Ji R.R., Acheson A., Lindsay R.M., Wilkinson G.A. (1999). Neurotrophins: Peripherally and centrally acting modulators of tactile stimulus-induced inflammatory pain hypersensitivity. Proc. Natl. Acad. Sci. USA.

[55] Grahl N., Shepardson K.M., Chung D., Cramer R.A. (2012). Hypoxia and fungal pathogenesis: To air or not to air?. Eukaryotic Cell.

[56] Ito T., Connett J.M., Kunkel S.L., Matsukawa A. (2012). Notch system in the linkage of innate and adaptive immunity. J. Leukoc. Biol..

[57] Burroughs S.K., Kaluz S., Wang D., Wang K., Van Meir E.G., Wang B. (2013). Hypoxia inducible factor pathway inhibitors as anticancer therapeutics. Future Med. Chem..

[58] Semenza G.L. (2003). Targeting HIF-1 for cancer therapy. Nat. Rev. Cancer.

[59] Jing X., Yang F., Shao C., Wei K., Xie M., Shen H., Shu Y. (2019). Role of hypoxia in cancer therapy by regulating the tumor microenvironment. Mol. Cancer.

[60] Rice P., Longden L., Bleasby A. (2000). EMBOSS: The European Molecular Biology Open Software Suite. Trends Genet..

[61] Lee H.T., Lee C.C., Yang J.R., Lai J.Z.C., Chang K.Y., Ray O. (2015). A large-scale structural classification of Antimicrobial peptides. BioMed Res. Int..

[62] Jhong J.H., Chi Y.H., Li W.C., Lin T.H., Huang K.Y., Lee T.Y. (2019). dbAMP: An integrated resource for exploring antimicrobial peptides with functional activities and physicochemical properties on transcriptome and proteome data. Nucleic Acids Res..

[63] Pirtskhalava M., Amstrong A.A., Grigolava M., Chubinidze M., Alimbarashvili E., Vishnepolsky B., Gabrielian A., Rosenthal A., Hurt D.E., Tartakovsky M. (2021). DBAASP v3: Database of antimicrobial/cytotoxic activity and structure of peptides as a resource for development of new therapeutics. Nucleic Acids Res..

[64] Lin W., Xu D. (2016). Imbalanced multi-label learning for identifying antimicrobial peptides and their functional types. Bioinformatics.

[65] Agrawal P., Bhalla S., Chaudhary K., Kumar R., Sharma M., Raghava G.P.S. (2018). In Silico Approach for Prediction of Antifungal Peptides. Front. Microbiol..

[66] Maingi V., Jain V., Bharatam P.V., Maiti P.K. (2012). Dendrimer building toolkit: Model building and characterization of various dendrimer architectures. J. Comput. Chem..

[67] Keiser M.J., Roth B.L., Armbruster B.N., Ernsberger P., Irwin J.J., Shoichet B.K. (2007). Relating protein pharmacology by ligand chemistry. Nat. Biotechnol..

[68] Daina A., Michielin O., Zoete V. (2019). SwissTargetPrediction: Updated data and new features for efficient prediction of protein targets of small molecules. Nucleic Acids Res..

[69] Lin G., Chai J., Yuan S., Mai C., Cai L., Murphy R.W., Zhou W., Luo J. (2016). VennPainter: A Tool for the Comparison and Identification of Candidate Genes Based on Venn Diagrams. PLoS ONE.

[70] Wang Y., Zhang S., Li F., Zhou Y., Zhang Y., Wang Z., Zhang R., Zhu J., Ren Y., Tan Y. (2020). Therapeutic target database 2020: Enriched resource for facilitating research and early development of targeted therapeutics. Nucleic Acids Res..

[71] Amberger J.S., Bocchini C.A., Scott A.F., Hamosh A. (2019). OMIM.org: Leveraging knowledge across phenotype-gene relationships. Nucleic Acids Res..

[72] Szklarczyk D., Gable A.L., Lyon D., Junge A., Wyder S., Huerta-Cepas J., Simonovic M., Doncheva N.T., Morris J.H., Bork P. (2019). STRING v11: Protein-protein association networks with increased coverage, supporting functional discovery in genome-wide experimental datasets. Nucleic Acids Res..

[73] O’Boyle N.M., Banck M., James C.A., Morley C., Vandermeersch T., Hutchison G.R. (2011). Open Babel: An Open chemical toolbox. J. Cheminform..

[74] Morris G.M., Ruth H., Lindstrom W., Sanner M.F., Belew R.K., Goodsell D.S., Olson A.J. (2009). Software news and updates AutoDock4 and AutoDockTools4: Automated docking with selective receptor flexibility. J. Comput. Chem..

[75] Khanal P., Patil B.M., Chand J., Naaz Y. (2020). Anthraquinone Derivatives as an Immune Booster and their Therapeutic Option Against COVID-19. Nat. Prod. Bioprospect..

[76] Laskowski R.A., Swindells M.B. (2011). LigPlot+: Multiple ligand-protein interaction diagrams for drug discovery. J. Chem. Inf. Model..

